# Joint modeling of longitudinal outcomes and survival using latent growth modeling approach in a mesothelioma trial

**DOI:** 10.1007/s10742-012-0092-z

**Published:** 2012-06-05

**Authors:** Ping Wang, Wei Shen, Mark Ernest Boye

**Affiliations:** Lilly Corporate Center, Eli Lilly and Company, Indianapolis, IN 46285 USA

**Keywords:** Joint modeling, Cox proportional hazards model, Latent trajectory, Random effects, Oncology clinical trial, Patient-reported outcomes

## Abstract

Joint modeling of longitudinal and survival data can provide more efficient and less biased estimates of treatment effects through accounting for the associations between these two data types. Sponsors of oncology clinical trials routinely and increasingly include patient-reported outcome (PRO) instruments to evaluate the effect of treatment on symptoms, functioning, and quality of life. Known publications of these trials typically do not include jointly modeled analyses and results. We formulated several joint models based on a latent growth model for longitudinal PRO data and a Cox proportional hazards model for survival data. The longitudinal and survival components were linked through either a latent growth trajectory or shared random effects. We applied these models to data from a randomized phase III oncology clinical trial in mesothelioma. We compared the results derived under different model specifications and showed that the use of joint modeling may result in improved estimates of the overall treatment effect.

## Introduction

Clinical research often generates both longitudinal and survival (time-to-event) data. Well-established methods exist for separately analyzing each type of data. For longitudinal data, mixed-effects models for repeated measures (MMRM) are often used, which can handle data that is missing at random. For survival data, semiparametric methods such as Cox proportional hazards models and parametric methods such as Weibull models are commonly used. Although useful, separate analyses of each type of outcome may not be able to provide adequate answers to some important research questions. One such example is whether CD4 lymphocyte count could serve as a good surrogate marker for clinical progression in AIDS clinical trials (Tsiatis et al. 1995). Another example is whether changes in the Positive and Negative Symptoms Scale, an instrument commonly used to assess disease status in patients with schizophrenia, are associated with the time to discontinuation of therapy (Henderson et al. 2000).

To answer these questions, methods for the combined analysis of the two types of data have been developed recently. A naive method is to incorporate the longitudinal measures directly into the Cox model as time-varying covariates. As noted by researchers (e.g., Tsiatis et al. 1995; Yu et al. 2004), this method does not account for measurement errors in the time-varying covariates and therefore can cause the estimated relative risk parameter in the time-dependent Cox model to be biased toward the null (Prentice 1982).

Tsiatis et al. (1995) proposed a two-stage approach to improve the naive method. In this approach, a linear mixed-effects model is fit to the longitudinal data, and then the fitted trajectory is incorporated into the Cox model as time-varying covariates. However, this approach has the potential for biased estimates when the longitudinal process is informatively censored at the event time (Hanson et al. 2011).

Disadvantages of the naive method and two-stage approach motivated the recent development of joint models for longitudinal and survival data (see Tsiatis and Davidian 2004 for a review). In joint models, there are two components: a longitudinal process and a survival process. For individual *i*, the longitudinal process, *Y*_*i*_(*t*), is modeled with an underlying latent process η_*i*_(*t*) and the deviations *e*_*i*_(*t*) due to the measurement error and biological variation, i.e., *Y*_*i*_(*t*) = η_*i*_(*t*) + *e*_*i*_(*t*). In this paper, we use latent growth models for longitudinal data given their strengths such as (1) occasions of measurement need not be equally spaced, (2) the models can account for both measured and unmeasured covariates, (3) the models can account for measurement errors, and (4) as in general structural equation models, statistical models are flexible. In the survival process, event time *T*_*i*_ depends on η_*i*_(*t*) or random effects included in η_*i*_(*t*) or both. Joint likelihoods are specified based on these two components, then estimation and inferences are made using frequentist (Wulfsohn and Tsiatis 1997; Henderson et al. 2000; Song et al. 2002) or Bayesian (Faucett and Thomas 1996; Xu and Zeger 2001; Wang and Taylor 2001; Brown and Ibrahim 2003; Guo and Carlin 2004; Hatfield et al. 2011) approaches.

Several advantages have been noted with joint modeling (Ibrahim et al. 2010): (1) It addresses the informative censoring induced from the absence of longitudinal observations beyond the event time (Muthén et al. 2009); (2) It reduces estimation biases by accounting for measurement error and informative censoring; (3) It may increase statistical efficiency by using all of the data simultaneously in a single model; (4) It allows inferences for all three aspects: the treatment effect on longitudinal process; the association between the longitudinal process and survival; and the treatment effect on survival including the direct treatment effect on survival, the indirect treatment effect on survival through the latent longitudinal process, and therefore the overall treatment effect on survival, which is the sum of the direct and indirect effect.

Sponsors of oncology clinical trials routinely and increasingly include patient-reported outcome (PRO) measures over time to evaluate the effect of treatment on symptoms, functioning, and quality of life (QoL). PRO data, along with tumor progression and overall survival rate, provide a comprehensive assessment of benefit and risk for treatment in late-stage cancer. Treatment that delays tumor progression is often associated with better symptoms and QoL progress. On the other hand, improvement in symptoms and QoL may serve as an indicator of a positive tumor response or lack of tumor progression. As an effective treatment often impacts both tumor progression/survival and symptoms simultaneously, it is important to understand the impact of a treatment on both outcomes and the association between two types of outcomes through joint modeling.

In this paper, we applied joint models to data from a randomized phase III oncology clinical trial in patients with malignant pleural mesothelioma (MPM) (Vogelzang et al. 2003). Researchers in this Vogelzang et al. (2003) study (also known as EMPHACIS) collected PRO measures throughout the course of treatment, using the patient-reported Lung Cancer Symptom Scale (LCSS) (Hollen et al. 1995). Previously, researchers have investigated the prognostic effect of baseline PRO measures on overall survival in patients with MPM (Bottomley et al. 2007). We however were interested in the association between post-baseline PRO scores and time to progressive disease (TTPD). The main goal of applying joint models in this study was to assess the treatment effect on LCSS symptoms and global measures of functioning and QoL, the association between the longitudinal LCSS items and TTPD, and the overall treatment effect on TTPD.

The remainder of this paper is organized as follows. In Sect. 2, we describe the EMPHACIS dataset. In Sect. 3, we formulated several joint models based on a latent growth model for longitudinal PRO data and a Cox proportional hazard model for survival data. The longitudinal and survival components were linked through either a latent growth trajectory or shared random effects. We adopted the path diagrams to visually represent the joint models; these diagrams help to illustrate the indirect and direct relationships among observed and latent variables. Section 4 presents results from our joint models applied to the EMPHACIS datasets with detailed interpretation. In addition, results from the joint modeling approach were compared with the results from separate modeling approaches. We conclude in Sect. 5 with a discussion of results and limitations of the joint modeling approach, and offer suggestions concerning model extensions.

## Motivating example, the MPM clinical trial

### Patients

EMPHACIS was a global phase III clinical trial conducted to evaluate the efficacy of pemetrexed/cisplatin, compared with cisplatin alone, as first-line treatment for patients with MPM (Vogelzang et al. 2003).

A total of 448 eligible patients were randomly assigned and received therapy (pemetrexed/cisplatin,* n* = 226; cisplatin alone,* n* = 222), and they were considered as the intent-to-treat (ITT) population. Pemetrexed/cisplatin or cisplatin was administered on day 1 of a 21-day cycle. A regimen of pemetrexed/cisplatin or cisplatin was defined as six cycles of therapy. A patient who was receiving benefit from treatment could receive additional cycles based on the discretion of the investigator. Treatment was discontinued for disease progression or intolerable toxicity, or on patient or investigator request. Pemetrexed/cisplatin patients received more treatment cycles (median, 6 cycles; range, 1–12 cycles) than those receiving cisplatin alone (median, 4 cycles; range, 1–9 cycles).

The TTPD was the time from randomization until a documented progression or death from any cause. For patients without progressive disease at the time of analysis, the date of the last follow-up was considered right-censored. Vogelzang et al. (2003) showed an increased TTPD of 1.8 months (median 5.7 months in pemetrexed/cisplatin versus 3.9 months in cisplatin alone).

### Patient-reported outcome measures

The PROs were measured with the well-established and validated LCSS, a nine-item instrument scored through responses that patients recorded on 100-mm visual analog scales, with zero representing absence of the symptoms or impairment, or high QoL, and 100 representing as much symptoms or impairment as there could be or low QoL. The nine scales represent six lung-cancer-related symptoms (anorexia, cough, dyspnea—shortness of breath, fatigue, hemoptysis, and pain) and three global measures (symptom distress, interference with carrying out normal activities, and global QoL). To be consistent with the LCSS validation studies in mesothelioma (Hollen et al. 2006), which indicated that hemoptysis was not a relevant symptom in patients with MPM, we excluded hemoptysis from our analyses. Accordingly, we included two average symptom burden indices (ASBIs) in our analyses: the ASBI5 (the mean of the five remaining symptom items: anorexia, cough, dyspnea, fatigue, and pain) and the ASBI8 (the mean of the five symptom items and the three global items).

The LCSS assessments were scheduled at two baseline visits (4–6 days and 1–2 days before the start of study drug therapy), weekly during the study (at days 8 ± 1, 15 ± 1, and 19 of each cycle), and approximately every 3 months after the patient had received his or her last dose of treatment if the patient had not initiated subsequent therapy. To eliminate intra-cycle variability in the LCSS scores and reduce computational burden, we calculated the mean of each patient’s scores for each LCSS item within each cycle. Accordingly, for each cycle the LCSS were assessed, we included the corresponding measurement time into the models as the mean number of days from randomization to LCSS assessments within the cycle.

Before disease progression or death, over 90 % of the patients completed LCSS assessments at each cycle per study protocol. Beyond disease progression, very few LCSS assessments were available. Given one of our primary interests was to assess the association between the TTPD and LCSS scores obtained prior to TTPD, we excluded the LCSS measurements obtained after tumor progression. In addition, considering that the protocol defined regimen was 6 cycles, we excluded the LCSS measurements obtained after cycle 6, which allowed us to focus on the treatment effect on the LCSS scores within the first 6 cycles. Only 20 (4.5 %) patients received more than 6 cycles of treatment; the exclusion of these data should have little impact on our results. Termination of LCSS measurements was closely related to TTPD, which introduced the problem of informative censoring.

## Methods

For individual *i*, let *Y*_*ij*_ be the observed longitudinal data at times $t_{ij}, j=0, \ldots, J_i$, and let η_*i*_(*t*) be the latent trajectory function underlying *Y*_*ij*_. Let *T*_*i*_ be the observed event time, which is the minimum of the event time *T*_*i*_^0^ and the censoring time *C*_*i*_. Let δ_*i*_ be the censoring indicator,

$$ \delta_i= \left\{\begin{array}{ll}  1, & \hbox{if} \,T^0_i{>}C_i, \hbox{censored} \\  0, & \hbox{if} \,T^0_i\leq C_i, \hbox{event}. \end{array} \right. $$

Let *Z*_*i*_ be the treatment indicator, *Z*_*i*_ = 1 if individual *i* received pemetrexed/cisplatin and *Z*_*i*_ = 0 if cisplatin was received, so that treatment effect refers to pemetrexed/cisplatin versus cisplatin.

### Separate analyses

Before looking into the joint models, we performed separate analyses for longitudinal data and survival data. When longitudinal data are incomplete, the MMRM is a commonly used method (Siddiqui et al. 2009). We included treatment, cycle, and treatment-by-cycle interaction as fixed effects and BIC was used to choose between compound symmetry and AR(1) covariance matrices in the MMRM analysis.

For the survival data, we fit the Cox proportional hazards model with treatment as the only covariate (Cox 1975), i.e.,

$$ \log h_i(t)=\log h_0(t)+\alpha Z_i, $$

where *h*_*i*_(*t*) and *h*_0_(*t*) are the hazard function for individual *i* and the baseline hazard function.

### Cox model with time-varying covariates

We also performed the combined analysis that incorporated the longitudinal measures directly into the Cox model as time-varying covariates. This method can be described as

$$ \log h_i(t)=\log h_0(t)+ {\user2{X}_{si}^{\prime}} {\varvec{\alpha}}+\gamma Y_i(t), $$

where ${\user2{X}_{si}^{\prime}}$ is the covariate vector affecting survival that may include treatment (*Z*_*i*_) and other covariates, ${\varvec{\alpha}}$ is the corresponding coefficient vector, and γ measures the association between longitudinal measures and survival. This naive method does not account for measurement errors of longitudinal outcome.

### Joint models for longitudinal data and survival data

Joint models have two linked components: the longitudinal component and the survival component. The longitudinal component consists of a model for longitudinal outcome, in which a trajectory function is often specified. The survival component consists of a model for survival data. We describe two types of joint models in which the longitudinal and survival components are linked differently. Both types of models use the following latent growth model to describe the longitudinal data,

1 $$ Y_{ij} =\eta_i(t_{ij})+\varepsilon_{ij} $$

2 $$ \eta_i(t) = {\user2{f}}(t)^T{\user2{b}_{\user2{i}}}\\ $$

3 $$ {\user2{b}_{\user2{i}}= {\varvec{\beta}} {\user2{X}_{i}}+{\varvec{\zeta}}_{i}} $$

where $\varepsilon_{ij} \sim N(0, \sigma^2)$ are mutually independent measurement errors, ${\user2{f}}(t)$ is a vector of functions of $t, {\user2{b}_{\user2{i}}}$ is an individual specific parameter vector (random effects), ${\varvec{\beta}}$ is a regression parameter matrix, and ${\varvec{\zeta_i}}$ are residuals following a multivariate normal distribution with mean zero and variance covariance matrix ${\varvec{\Upsigma}}_{r\times r}$.

The formulation of the longitudinal model above offers great flexibility and links well to the commonly used mixed-effects models. For example, consider a mixed-effects model, $Y_{ij}=\beta_{00}+\beta_{10}t_{ij}+\beta_{11}Z_i t_{ij} +\zeta_{0i}+\zeta_{1i}t_{ij}+\varepsilon_{ij},$ where the fixed-effects part is β_00_ + β_10_ *t*_*ij*_ + β_11_ *Z*_*i*_ *t*_*ij*_, assuming a treatment-by-time interaction, and the random-effects part is $\zeta_{0i}+\zeta_{1i}t_{ij}$. This model can be easily written in the latent growth model format with the following specification:

$$ {\user2{f}}(t)=(1, t)', \quad {\user2{b}_{\user2{i}}}= (b_{0i}, b_{1i})', \quad {\varvec{\beta}}= \left(\begin{array}{cc} \beta_{00} & 0 \\ \beta_{10} & \beta_{11} \\ \end{array} \right), \quad {\user2{X}_{\user2{i}}} = (1, Z_i)', \quad {\varvec{\zeta}_{i}}= (\zeta_{0i}, \zeta_{1i}).' $$

This model is introduced as l_s below.

In our analysis, we considered five choices of η_*i*_(*t*) depending on the shape of the trajectory function and the treatment effect associated with the trajectory function. The first three choices, named l_s, l_i, and l_is assume a linear growth for each LCSS item, i.e., η_*i*_(*t*) = *b*_0*i*_ + *b*_1*i*_ *t*. In addition, model l_s assumes treatment effect on slope only ($b_{0i}=\beta_{00} + \zeta_{0i}, b_{1i}=\beta_{10}+\beta_{11}Z_i+\zeta_{1i}$); model l_i assumes treatment effect on intercept only; and model l_is assumes treatment effect on both intercept and slope. The other two choices, named q_s and q_sq, assume a quadratic growth for each LCSS item, i.e., η_*i*_(*t*) = *b*_0*i*_ + *b*_1*i*_ *t* + *b*_2*i*_ *t* ^2^. Model q_s assumes treatment effect on slope only ($b_{0i}=\beta_{00} + \zeta_{0i}, b_{1i}=\beta_{10} + \beta_{11}Z_i+\zeta_{1i},$ $b_{2i}=\beta_{20}+\zeta_{2i}$) and model q_sq assumes treatment effect on both slope and quadratic coefficient. In models l_s, l_is, q_s, and q_sq, where a treatment effect on slope is assumed, a treatment-by-time interaction is explicitly assumed. We checked the treatment effect on the intercept in the two linear growth models although we expect that there is no difference at baseline across treatment groups for this randomized trial.

#### Trajectory model

In the trajectory model, the longitudinal and survival components are linked through the latent trajectory. For example, Xu and Zeger (2001) proposed to use a Markov Chain Monte Carlo algorithm to estimate the posterior distribution for parameters in the joint model in which the survival component consists of

4 $$ \log{h_i(t)}=\log{h_0(t)}+{\user2{X}_{si}^{\prime}}{\varvec{\alpha}}+ \gamma \eta_i(t), $$

where γ measures the association between survival and the trajectory η_*i*_(*t*) that varies continuously over time.

An alternative way to describe the survival component was proposed by Asparouhov et al. (2006) and is detailed here. First, the time interval is split into subintervals [*t*_*k*−1_, *t*_*k*_), *k* = 1, 2,…,*K*, *t*_0_ = 0, $t_K=\infty,$ and a separate survival variable *T*_*ik*_ and censoring indicator δ_*ik*_ are created for each subinterval [*t*_*k*−1_, *t*_*k*_) from the original survival variable *T*_*i*_ and censoring indicator δ_*i*_ as follows:

5 $$ T_{ik}= \left\{\begin{array}{ll} t_k-t_{k-1}, &\hbox{if}\, t_k < T_i,\\ \hbox{missing}, & \hbox{if}\, T_i<t_{k-1}, \\ T_i-t_{k-1}, & \hbox{otherwise}; \end{array} \right. \quad \delta_{ik}= \left\{ \begin{array}{ll} 1, & \hbox{if} \,t_k<T_i, \\ \hbox{missing}, & \hbox{if}\, T_i<t_{k-1}, \\ \delta_i, & \hbox{otherwise}. \end{array}\right. $$

Then, for *t* in the time interval [*t*_*k*−1_, *t*_*k*_), 

6 $$ \log h_{ik}(t)=\log h_0(t)+ {\user2{X}_{si}^{\prime}} {\varvec{\alpha}}+\gamma \eta_i(t_{k-1}), $$

*h*_0_(*t*) is a non-parametric baseline hazard function, and the likelihood for the survival variable *t*_*Ki*-1_ ≤ *T*_*i*_ < *t*_*Ki*_ is

7 $$ p(T_i, \delta_i|{\user2{b}_{\user2{i}}}; {\varvec{\alpha}}, \gamma)=\prod_{k=1}^{K_i}h_{ik}(T_{ik})^{(1-\delta_{ik})} \hbox{exp} \left\{-\sum_{k=1}^{K_i}\int_{t_{k-1}}^{t_{k-1}+T_{ik}} h_{ik}(s)ds\right\}. $$

This model uses the stepwise predictor η_*i*_(*t*_*k*−1_) and is estimated by the maximum likelihood algorithm. If the step size is chosen to be sufficiently small, the difference between this model and the model described by Xu and Zeger (2001) will be negligible.

**Fig. 1:**
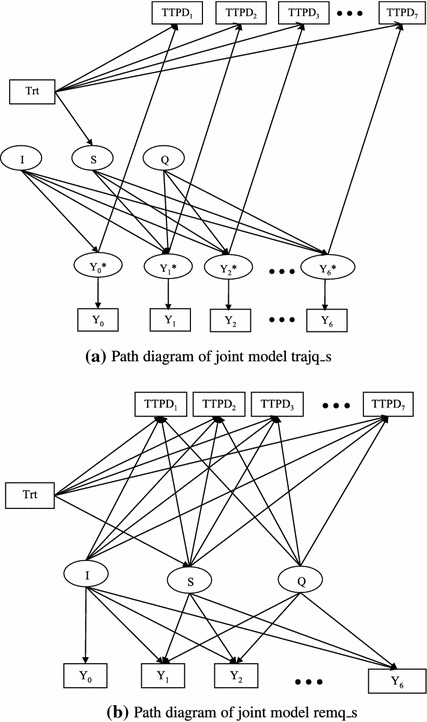
Path diagrams of joint models trajq_s and remq_s.* Trt* treatment;* I* intercept (*b*_0*i*_);* S* slope (*b*_1*i*_);* Q* quadratic coefficient (*b*_2*i*_); $y_{k}^{\ast}$ = η(*t*_*k*_)

We used the model proposed by Asparouhov et al. (2006) in this paper. When applying this model to the EMPHACIS trial data, we let $t_k=0.7k, k=1, 2, \ldots, 7,$ because each cycle was 0.7 month. We considered five trajectory models with (5–6) for the survival component and the models l_i, l_s, l_is, q_s, and q_sq for the longitudinal component. These models are named by adding the prefix “traj” to the corresponding longitudinal model name. The path diagram in Fig. 1a represents the trajectory model trajq_s. In the figure, the rectangles represent the observed variables, the ellipses represent latent variables (I for intercept, S for slope, Q for quadratic coefficient, and $Y_{k}^{\ast}$ = η(*t*_*k*_)), and the arrows point to the dependent variables.

#### Shared random-effects model

In the shared random-effects model, the longitudinal component and survival component are linked through the random-effects ${\user2{b}_{\user2{i}}}$:

8 $$ \log h_i(t) =\log{h_0(t)}+{\user2{X}_{si}^{\prime}} {\varvec{\alpha}}+{\user2{b}_{i}^{\prime}} {\varvec{\gamma}}. $$

Similar to the trajectory models, we considered five choices for the longitudinal component (l_i, l_s, l_is, q_s, and q_sq), along with (8) for the survival component. These models are named by adding the prefix “rem” to the corresponding longitudinal model name. The path diagram in Fig. 1b represents the random-effects model remq_s:

$$ \eta_i(t)=b_{0i}+b_{1i}t+b_{2i}t^2, \quad b_{0i}=\beta_{00} + \zeta_{0i}, \quad b_{1i}=\beta_{10}+\beta_{11}Z_i+\zeta_{1i}, \quad b_{2i}=\beta_{20} + \zeta_{2i}, $$

$$ \log{h_i(t)} =\log{h_0(t)}+\alpha Z_i+\gamma_0 b_{0i}+\gamma_1 b_{1i}+\gamma_2 b_{2i}, $$

and *h*_0_(*t*) is a non-parametric baseline hazard function. In this setting, it is easy to see a direct treatment effect (α) on TTPD and an indirect treatment effect (γ_1_β_11_) on TTPD through random slopes (*b*_1*i*_). Combining the direct and indirect treatment effects, we derived the overall treatment effect on TTPD as α + γ_1_β_11_. The decomposition of the overall effect into the direct and indirect effects can be generalized to a non-treatment covariate if it is incorporated into both the longitudinal and survival components of the joint model. This feature represents an important advantage of the shared random-effects model.

We used the software Mplus Version 6 (Muthén and Muthén 1998–2010) to fit the trajectory models and shared random-effect models using maximum likelihood estimation. Different joint models were compared using the Bayesian Information Criterion (BIC) (Schwarz 1978) defined as

$$ BIC=-2\log L({\varvec{\alpha}}, {\varvec{\gamma}}, \sigma, {\varvec{\beta}}, {\varvec{\Upsigma}})+p\log(n), $$

where *p* is the number of parameters in the model, *n* is the sample size, and the joint log-likelihood is given by:

$$ \begin{aligned} \log L({\varvec{\alpha}}, {\varvec{\gamma}}, \sigma, {\varvec{\beta}}, {\varvec{\Upsigma}}) &= \sum_{i=1}^{n} \log \int p(T_i, \delta_i|{\user2{b}}_i; {\varvec{\alpha}}, {\varvec{\gamma}}) \prod_{j=1}^{J_i}p(Y_{ij}|{\user2{b}}_i; \sigma)p({\user2{b}}_i| {\varvec{\beta}}, {\varvec{\Upsigma}}) d{\user2{b}}_i, \\ p(Y_{ij}|{\user2{b}}_i; \sigma) &= \frac{1}{\sqrt{2\pi\sigma^2}} \exp\left\{-\frac{(Y_{ij}-{\user2{f}}(t_{ij})^T{\user2{b}}_i)^2} {2\pi\sigma^2}\right\}, \\ p({\user2{b}}_i|{\varvec{\beta}}, {\varvec{\Upsigma}}) &= \frac{1}{(2\pi)^{r/2}|{\varvec{\Upsigma}}|^{1/2}} \exp\left\{-\frac{1}{2}({\user2{b}}_i- {\varvec{\beta}}{\user2{X}}_i)^T {\varvec{\Upsigma}}^{-1}({\user2{b}}_i- {\varvec{\beta}}{\user2{X}}_i)\right\}. \\ \end{aligned} $$

In the trajectory model, $p(T_i, \delta_i|{\user2{b}_{\user2{i}}}; {\varvec{\alpha}}, \gamma)$ is given in (7), and in the shared random-effect model,

$$ p(T_i, \delta_i|{\user2{b}_{\user2{i}}}; {\varvec{\alpha}}, {\varvec{\gamma}})=h_i(T_i)^{(1-\delta_i)} \hbox{exp}\left\{-\int_{0}^{T_i} h_i(s)ds\right\} $$

with $h_i(\cdot)$ given by (8).

The BIC is readily available in Mplus for both trajectory and random-effects models. The model with a lower value of BIC is preferred. There are many other approaches for model selection. For example, researchers using Bayesian approaches for joint modeling often use the Deviance Information Criterion (DIC) (Spiegelhalter et al. 2002). Guo and Carlin (2004) and Hatfield et al. (2011) used the DIC for model selection in joint modeling and provided rich discussions on the DIC.

## Results

### Separate analyses

In the MMRM analyses, we used both compound symmetry and AR(1) covariance matrices. Because the MMRM analyses with AR(1) gave smaller BIC, we reported the results using the AR(1) covariance matrix in Table 1. The least square mean (LSMean) and standard error (SE) of the LSMean for each LCSS item in each treatment arm at cycle 6 are reported, as well as the * P*-value for testing the difference in LCSS mean score between the two treatment arms at cycle 6. Significantly (or trending to significantly) lower mean scores in pemetrexed/cisplatin were observed on dyspnea, ASBI5, and ASBI8 (*P*-values <0.1).

**Table 1:** Least square means (LSMean) at cycle 6 from the mixed-effects model for repeated measures (MMRM) analysis

LCSS item	Cisplatin		Pemetrexed/cisplatin		Difference
	LSMean	SE	LSMean	SE	*P*-value
Anorexia	32.4	2.2	32.3	1.9	0.961
Cough	11.8	1.5	10.5	1.3	0.523
Dyspnea	36.9	2.0	30.9	1.7	0.023
Fatigue	44.7	2.1	41.3	1.8	0.218
Pain	28.7	2.0	25.0	1.7	0.157
Interference	48.5	2.0	44.5	1.8	0.136
QoL	47.3	2.0	43.6	1.7	0.164
Symptoms	38.9	2.0	35.4	1.7	0.194
ASBI5	31.6	1.4	28.3	1.2	0.076
ASBI8	36.9	1.4	33.2	1.3	0.053

*LCSS* Lung Cancer Symptom Scale,* ASBI5* the mean of the five symptom items (anorexia, cough, dyspnea, fatigue, and pain),* ASBI8* the mean of the five symptom items and the three global items (interference, QoL, and symptoms).* SE* standard error

For survival data, we fitted the Cox proportional hazards model with treatment as the only covariate and the hazard ratio (HR) for treatment (pemetrexed/cisplatin versus cisplatin) was HR_0_ = 0.73 (*P*-value = 0.001).

### Cox model with time-varying covariates

The naive method was applied, i.e., each LCSS item was incorporated in the Cox model as a time-varying covariate. The HRs for a one-point increase in LCSS items and the HRs for treatment are presented in Table 3 under “Naive Model”. The* P*-values for these HRs were all less than 0.01, indicating significant effects of both LCSS and treatment on TTPD. The hazard increased approximately from 9 % (cough) to 23 % (ASBI5), with a 10-point increase in the observed LCSS score.

### Joint models without covariates other than treatment

We jointly modeled TTPD with each LCSS item using the five trajectory models and the five random-effects models described in Sect. 3. Treatment was the only covariate we considered in both longitudinal and survival components. There were no significant treatment effects on random intercepts in the trajl_i, trajl_is, reml_i, and reml_is models (all* P*-values $\geqslant 0.05$ except for anorexia in the reml_is with* P*-value = 0.039). Under the trajectory model framework, the trajq_s models gave the smallest BIC. Table 2 lists BIC for the trajq_s models, and BIC differences between the other four trajectory models and the trajq_s model. Under the random-effects model framework, again using the model q_s for the longitudinal component led to the smallest BIC. Table 2 lists BIC for the remq_s model, and BIC differences between the other four random-effects models and the remq_s model. According to Raftery (1995), under either the trajectory or the random-effects model framework, the BIC difference between q_s and q_sq represents positive evidence favoring q_s, and the BIC difference between q_s and the three l models represents very strong evidence favoring q_s (difference of 0–2, 2–6, 6–10, and >10 represents weak, positive, strong, and very strong evidence, respectively, of favoring the model with smaller BIC). Therefore, we chose to present the detailed results from the best models under each framework, trajq_s and remq_s, in Tables 3 and 4, respectively.

**Table 2:** BIC and BIC differences for joint models

	Anorexia	Cough	Dyspnea	Fatigue	Pain	Interference	QoL	Symptoms	ASBI5	ASBI8
BIC(trajq_s)	21,139	19,805	20,745	21,050	20,794	20,823	20,697	20,654	19,013	19,145
BIC(trajl_i)-BIC(trajq_s)	69	40	98	77	108	68	71	113	113	100
BIC(trajl_s)-BIC(trajq_s)	72	39	95	74	103	68	70	113	108	97
BIC(trajl_is)-BIC(trajq_s)	74	43	101	79	109	72	76	116	113	101
BIC(trajq_sq)-BIC(trajq_s)	6	6	6	6	1	5	5	6	6	6
BIC(remq_s)	21,147	19,818	20,753	21,056	20,800	20,827	20,703	20,662	19,020	19,150
BIC(reml_i)-BIC(remq_s)	59	35	89	67	101	55	60	105	98	84
BIC(reml_s)-BIC(remq_s)	61	34	85	63	94	54	59	104	92	80
BIC(reml_is)-BIC(remq_s)	63	37	91	68	99	58	65	107	96	84
BIC(remq_sq)-BIC(remq_s)	5	6	5	6	3	5	4	6	6	6

*ASBI5* the mean of the five symptom items (anorexia, cough, dyspnea, fatigue, and pain), *ASBI8* the mean of the five symptom items and the three global items (interference, QoL, and symptoms)

**Table 3:** Results from the naive method and joint model trajq_s

LCSS Item	Naive model		Joint Model Trajq_s					
	HR (LCSS)	HR (Trt)	β_00_	β_10_	β_11_	β_20_	*e* ^γ^: HR (LCSS)	*e* ^α^: HR (Trt)
Anorexia	1.011	0.67	28.8	5.93	−0.138	−1.15	1.014	0.66
Cough	1.009	0.72	14.7	−1.29	−0.410	0.193	1.015	0.72
Dyspnea	1.010	0.73	32.6	2.74	−1.280*	−0.327	1.011	0.73
Fatigue	1.012	0.70	36.4	6.62	−1.033	−1.032	1.014	0.70
Pain	1.016	0.70	26.8	0.63	−0.891	0.061	1.018	0.71
Interference	1.013	0.66	41.5	5.87	−0.881	−0.905	1.015	0.66
QoL	1.012	0.69	41.1	4.86	−0.873	−0.710	1.014	0.69
Symptoms	1.012	0.68	34.1	3.08	−0.805	−0.432	1.014	0.68
ASBI5	1.021	0.69	27.9	3.09	−0.866	−0.435	1.025	0.68
ASBI8	1.019	0.67	32.0	3.72	−0.883	−0.516	1.022	0.67

*LCSS* Lung Cancer Symptom Scale,* ASBI5* the mean of the five symptom items (anorexia, cough, dyspnea, fatigue, and pain),* ASBI8* the mean of the five symptom items and the three global items (interference, QoL, and symptoms),* HR* hazard ratio,* Trt* treatment

β_00_: mean of intercepts *b*_0*i*_ in LCSS trajectory η_*i*_(*t*) = *b*_0*i*_ + *b*_1*i*_ *t* + *b*_2*i*_ *t* ^2^

β_10_: mean of slopes *b*_1*i*_ for patients treated with cisplatin

β_11_: treatment effect on *b*_1*i*_; *P-value <0.05

β_10_ + β_11_: mean of slopes *b*_1*i*_ for patients treated with pemetrexed/cisplatin

β_20_: mean of quadratic coefficient *b*_2*i*_

γ: the association between LCSS trajectory and time to progressive disease (TTPD)

*P*-values for γ and α were less than 0.01

**Table 4:** Results from the remq_s model

LCSS item	β_00_	β_10_	β_11_	β_20_	γ_0_	γ_1_	γ_2_	*e* ^α^	$e^{\alpha+\gamma_1\beta_{11}}$
Anorexia	28.8	6.09	−0.37	−1.12	0.018**	0.060	0.293	0.66	0.65
Cough	14.7	−1.24	−0.62	0.26	0.017**	0.083**	0.359**	0.74	0.70
Dyspnea	32.6	2.89	−1.60*	−0.25	0.010**	0.064**	0.260**	0.76	0.68
Fatigue	36.3	6.92	−1.29	−1.03	0.014**	0.041	0.104	0.69	0.65
Pain	26.8	0.84	−1.30*	0.16	0.017**	0.087**	0.333**	0.74	0.66
Interference	41.5	6.08	−1.12	−0.88	0.017**	0.040	0.127	0.65	0.62
QoL	41.1	5.05	−1.06	−0.70	0.015**	0.032	0.070	0.67	0.65
Symptoms	34.1	3.26	−1.08	−0.39	0.015**	0.059*	0.219	0.69	0.65
ASBI5	27.9	3.23	−1.17*	−0.35	0.024**	0.105**	0.414**	0.71	0.63
ASBI8	32.0	3.86	−1.14*	−0.45	0.022**	0.086**	0.317**	0.69	0.62

*LCSS* Lung Cancer Symptom Scale,* ASBI5* the mean of the five symptom items (anorexia, cough, dyspnea, fatigue, and pain),* ASBI8* the mean of the five symptom items and the three global items (interference, QoL, and symptoms). β’s have the same meanings as those in Table 3. γ_0_, γ_1_, γ_2_ measure the association between random features of the LCSS trajectory and time to progressive disease (TTPD)

In columns β_11_, γ_0_, γ_1_, and γ_2_, * *P*-value <0.05, **P-value <0.01

*e* ^α^ is the direct treatment effect (pemetrexed/cisplatin versus cisplatin) in hazard ratio and $e^{\alpha+\gamma_1\beta_{11}}$ is the overall treatment effect in hazard ratio.* P*-values <0.01 for the direct treatment effects and* P*-values <0.001 for the overall treatment effects

Table 3 shows a significant treatment effect on slope of dyspnea (*P*-value = 0.039) only, trending to a significant treatment effect on slope of ASBI5 (*P*-value = 0.072) and ASBI8 (*P*-value = 0.079). When comparing the HRs of treatment from the trajq_s model (column *e* ^α^) with those from the naive method, the differences were minor. When comparing the HRs of the LCSS items, those from trajq_s (column *e* ^γ^) were all bigger than those from the naive method. Both phenomena are consistent with the findings in Ibrahim et al. (2010). Through simulation, they showed that the naive model and the joint model give nearly unbiased estimates for the direct treatment effect on survival (i.e., α here) and the naive model gives the biased estimate towards the null for the association between the longitudinal process and survival (i.e., γ here).

**Fig. 2:**
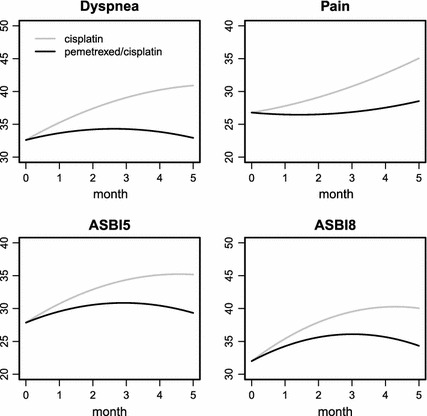
Fitted population-level quadratic growth curve of LCSS scores in the remq_s model. The cisplatin group is in* gray*, and the pemetrexed/cisplatin group is in* black*

Table 4 provides the parameter estimates for the other joint model, remq_s. There was a significant treatment effect on slope of dyspnea, pain, ASBI5, and ASBI8 (*P*-values for β_11_ were less than 0.05). On the one hand, this finding indicates that there was a significant treatment-by-time interaction effect on the growth of dyspnea, pain, ASBI5, and ASBI8. On the other hand, because the difference in the mean LCSS scores between the pemetrexed/cisplatin and cisplatin arms at time point *t* was β_11_ *t* under model remq_s, we could infer that patients in the pemetrexed/cisplatin arm had a significantly lower score on dyspnea, pain, ASBI5, and ASBI8 at cycle 6. This is similar with the findings in the MMRM analysis, in which significant or trending to significant treatment effects at cycle 6 were found on dyspnea, ASBI5, and ASBI8. From the parameter estimates of (β_00_, β_10_, β_11_, β_20_), we plotted the fitted mean quadratic growth curves under each treatment arm in Fig. 2 for the four items with a significant treatment effect on slope. The growth curves for pemetrexed/cisplatin are beneath those for cisplatin for all ten items; this finding was expected because the estimates for β_11_ were all negative. These curves show a beneficial effect of pemetrexed/cisplatin on the progress of the LCSS items.

In Table 4, γ_0_, γ_1_, and γ_2_ measure the association between the random features *b*_0*i*_, *b*_1*i*_, and *b*_2*i*_ of the LCSS trajectory and TTPD. For cough, dyspnea, pain, ASBI5, and ASBI8, all three features were significantly associated with TTPD (*P*-values for γ_0_, γ_1_ and γ_2_ were less than 0.01). For symptoms, two features were significantly associated with TTPD (*P*-value <0.01 for γ_0_ and *P*-value <0.05 for γ_1_). For the other four items, only the intercept of the trajectory was significantly associated with TTPD (*P*-value <0.01 for γ_0_). Joint tests on null hypothesis *H*_0_: γ_0_ = γ_1_ = γ_2_ = 0 were performed using Wald-tests; the *P*-values were all less than 0.001.

The overall treatment effects in terms of HRs are listed in column $e^{\alpha+\gamma_1\beta_{11}}$ in Table 4. They are consistently smaller than HR_0_ = 0.73 (i.e., the treatment effect without incorporating any longitudinal LCSS item), even when the treatment effect on LCSS was not significant. This finding is consistent with those by Ibrahim et al. (2010) and Chen et al. (2011).

### Joint model with other covariates

In the analyses presented in Sect. 4.3, we did not incorporate any covariates other than treatment. In this section, we considered six covariates in the model remq_s: bf for B12 and folic acid supplementation before treatment (bf = 1 if fully supplemented; bf = 0 if never or partially supplemented), kpsb for baseline Karnofsky performance status (KPS) (kpsb = 1 if baseline KPS = 90 or 100; kpsb = 0 if baseline KPS = 70 or 80), stagele3 for tumor stage (stagele3 = 1 if tumor stage is III or less; stagele3 = 0 if tumor stage is IV), agelt65 for age group (agelt65 = 1 if age <65 years old; agelt65 = 0 if age $\geqslant65$ years old), gender (1 if male, 0 if female) and race (1 if Caucasian, 0 if other). We did not include treatment and bf in the random intercept sub-model because the intercept represents the baseline status and is not impacted by post-baseline interventions, such as administration of treatments or supplementation of B12 and folic acid.

**Fig. 3:**
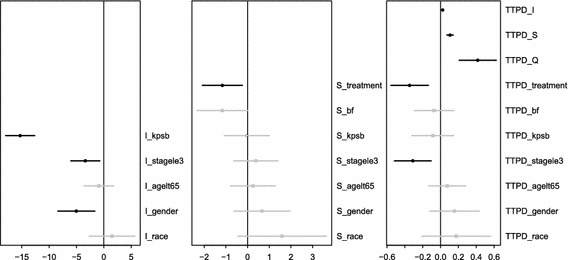
Parameter estimates and 95 % confidence intervals for effects of covariates on random intercepts, random slopes, and TTPD in the remq_s model for ASBI5 with covariates. Confidence intervals that exclude the null value of 0 are in* black*

The parameter estimates and their 95 % confidence intervals from the joint model of ASBI5 are summarized in Fig. 3. From the left plot for the sub-model of the random intercept (*b*_0*i*_), we see a significantly smaller intercept of ASBI5 (i.e. less symptoms at baseline) in men than women, in patients who have better performance status than those have worse performance status, and in patients who have tumor stage III or less at baseline than those have tumor stage IV. The middle plot for the sub-model of random slope (*b*_1*i*_) shows that patients receiving pemetrexed/cisplatin had significantly smaller slopes (i.e., slower worsening or quicker improving). The right plot for the sub-model of TTPD shows that all three random effects have significantly positive associations with the hazard and pemetrexed/cisplatin and stage III or less were significantly associated with improved TTPD.

By looking at the same plots for other items, several common findings are found: (1) patients with better performance status at baseline had significantly smaller intercepts of the item; (2) At least one random effect showed a significant positive association with the hazard; and (3) Pemetrexed/cisplatin and stage III or less were significantly associated with improved TTPD.

Similar to deriving the overall treatment effect on TTPD, we were able to derive the overall effect of other covariates from the joint model. Table 5 lists the direct and overall effects of treatment, kpsb, and stagele3 on TTPD. For every item, the overall kpsb effect was much larger (i.e., had a lower HR) than its direct effect. This was expected because kpsb had a significant effect on the intercept and the intercept was significantly associated with TTPD for every item. Thus, the indirect effect of kpsb on TTPD through intercept was big. In contrast, the direct and overall stagele3 effects were similar. The overall treatment effects were very similar to those from the remq_s model without covariates (see Table 4). Given this is a randomized study, this was not surprising. However, in a non-randomized observational study, joint models with covariates may be necessary to reduce bias.

**Table 5:** Direct and overall effects of treatment, kpsb, and stagele3 on TTPD in terms of hazard ratios

LCSS item	Treatment		kpsb		stagele3	
	Direct	Overall	Direct	Overall	Direct	Overall
Anorexia	0.65	0.64	0.88	0.69	0.77	0.72
Cough	0.72	0.68	0.78	0.71	0.72	0.73
Dyspnea	0.74	0.67	0.84	0.69	0.71	0.72
Fatigue	0.68	0.64	0.85	0.67	0.73	0.72
Pain	0.72	0.65	0.81	0.65	0.75	0.70
Interference	0.65	0.62	0.95	0.66	0.75	0.72
QoL	0.67	0.65	0.88	0.66	0.73	0.73
Symptoms	0.68	0.64	0.87	0.65	0.77	0.74
ASBI5	0.71	0.62	0.92	0.64	0.73	0.71
ASBI8	0.69	0.62	0.94	0.63	0.74	0.71

*Kpsb* baseline Karnofsky performance status (KPS) (kpsb = 1 if baseline KPS = 90 or 100; kpsb = 0 if baseline KPS = 70 or 80); stagele3 tumor stage (stagele3 = 1 if tumor stage is III or less; stagele3 0 if tumor stage is IV),* TTPD* time to progressive disease,* LCSS* Lung Cancer Symptom Scale,* ASBI5* the mean of the five symptom items (anorexia, cough, dyspnea, fatigue, and pain),* ASBI8* the mean of the five symptom items and the three global items (interference, QoL, and symptoms)

## Discussion

In this paper, we applied the joint modeling approach to an analysis of longitudinal PRO and survival outcomes from a clinical trial in patients with mesothelioma. Joint models allow us to simultaneously assess treatment effect on both longitudinal PRO and survival outcomes, as well as the association between these two outcomes. Our joint models produced different and seemingly more accurate results compared with models focused on PROs alone, or survival alone, or the naive model ignoring measurement errors in PROs by directly handling informative censoring and accounting for measurement errors. In addition, our joint models allowed specific modeling of PROs with latent trajectory and linked PROs and TTPD either through a latent trajectory or shared random effects. We compared our joint modeling approach with several standard approaches that analyzed longitudinal and survival data separately. Our joint models not only suggested a beneficial treatment effect on nearly all PRO measures at the end of the treatment period, but also were able to describe patterns (or trajectories) in PROs throughout the treatment period. Given the large treatment effect observed on TTPD in this study, both separate and joint modeling approaches showed a significant treatment effect on TTPD. However, the treatment effect on TTPD appeared to be larger in the joint models, which represented findings consistent with Ibrahim et al. (2010) and Chen et al. (2011). These authors showed that when the longitudinal data are associated with treatment, ignoring the longitudinal data in the survival model will lead to a biased estimate of the overall treatment effect on survival. In addition, our joint models allowed us to quantify the direct treatment effect on TTPD, as well as the indirect treatment effect through PROs.

A few specific features of the joint models deserve further discussions. First, in our longitudinal model for PROs, we explored both linear and non-linear trajectories (quadratic curves). Indeed, data from the mesothelioma trial suggested that the non-linear trajectory might be a better choice. For all the PRO measures with the exception of cough and pain, the fitted trajectories showed that PROs appeared to be worse during the first 3 months or the first four cycles for both treatment groups, and remained the same or improved after that. The initial worsening of PROs could be related to a large proportion of patients experiencing tumor progressions early on during the treatment, and/or the toxicity associated with the treatment. The rebound of PROs after 3 months suggested that those patients who were alive without tumor progression might have benefited from the treatment either in tumor response or improved tolerability. Further explorations are necessary to clarify this. Second, we used different functions to link the longitudinal and survival data: the trajectory function itself or shared random effects (such as slope in the trajectory function). In our analysis, use of either link function provided similar results (Tables 3, 4) as long as the shape of the growth curves was specified as quadratic. Guo and Carlin (2004) compared several link functions in their joint models, and showed that linking intercept (initial CD4 level) and the rate of CD4 decrease (slope) to survival provided a better fit in their analysis of data from AIDS clinical trials. However, their analyses were limited to a linear growth curve for longitudinal data. In the presence of a non-linear growth curve, use of the trajectory function as the link might provide a simple interpretation that survival is influenced by the current value of the longitudinal outcomes. Third, we showed that important baseline covariates could be included in our joint model. Our analysis confirmed the well-known association between performance status and PROs as well as the association between the stage of tumor and TTPD. Both performance status and the stage of tumor are important prognostic factors for TTPD and overall survival. In the setting of our clinical trials, both performance status and the stage of tumor were included as stratification variables in the randomization scheme, so treatment effects remained the same in the joint model including covariates. However, in a non-randomized observational study, joint models with covariates may be necessary to reduce bias.

While we have carefully considered our models and analyses, our work has several limitations. We have modeled each PRO item with TTPD one at a time. While this helped to establish the association between each PRO item and TTPD, often a treatment impacts multiple dimensions of PRO simultaneously, and changes in various PRO dimensions are related. An alternative is to develop a multivariate longitudinal model for all PRO items and link it to the survival model or reduce PROs to a single score, similar to the use of ASBI in our analysis. In either part of our joint models, assumptions may be violated either due to skewness of the PRO data with an excessive amount of zero data (i.e., absence of symptoms) or non-proportional hazards. Several modifications may lead to improvement in the performance of the joint models. For the longitudinal model, one may transform the PRO scores by a square-root transformation (Ibrahim et al. 2010), use a zero-inflated beta model (Hatfield et al. 2011), or model the change in PROs from baseline, which is more likely to be normally distributed. For the survival model, we may use alternatives to proportional hazards models, such as piece-wise exponential or parametric models.

Currently, no standard software exists to fit a wide range of joint models. Indeed, this presents a computational challenge, impeding the broader use of joint models in practice. Several authors have developed models that can be implemented in SAS$\circledR$ or WinBUGS (Guo and Carlin 2004; Ibrahim et al. 2010). An R package JM was recently developed for joint modeling of longitudinal and time-to-event data (Rizopoulos 2010). We chose Mplus to implement our models. Besides the trajectory and the shared random-effects models, other possibilities for joint modeling to meet different inference needs can also been implemented in Mplus, such as predicting survival from growth mixture (Muthén et al. 2009). While Mplus offered great flexibility in modeling longitudinal data with latent variables (such as the latent growth model, the latent class model, and the growth mixture model), several modifications are necessary in order to incorporate these longitudinal models into the survival model (e.g., creating survival variables on subintervals in joint models when using the trajectory function as the link). In general, we found most of our models and their extensions could be easily fit by Mplus using a frequentist-based computational algorithm. The Bayesian package for Mplus is currently under development.

As joint models become more commonly used for analyzing multiple clinical outcomes, there are abundant research opportunities for future work. One that potentially has a significant impact on clinical research and regulatory importance is the examination of the association between intermediate outcomes (such as TTPD and PROs) and ultimate outcomes (such as overall survival). A three-way joint model linking TTPD, PRO changes, and overall survival could be developed (Asparouhov et al. 2006). As various options for joint models exist, efficient and robust model selection criteria need to be developed in order to build the best joint models in both Bayesian and frequentist settings. Finally, our joint models offer a flexible framework in modeling multiple outcomes from clinical research. The idea of borrowing information across outcome types (such as efficacy and safety) through carefully selected fixed or random effects is easily generalizable.
